# Arrest of Viral Proliferation by Ectopic Copies of Its Cognate Replication Origin

**DOI:** 10.3390/genes6020436

**Published:** 2015-06-23

**Authors:** Manuel S. Valenzuela, Chakradhari Sharan

**Affiliations:** 1 Department of Biochemistry and Cancer Biology, School of Medicine, Meharry Medical College, 1005 D.B. Todd Jr. Boulevard, Nashville, TN 37208, USA; 2 Department of Obstetrics and Gynecology, School of Medicine, Meharry Medical College, 1005 D.B. Todd Jr. Boulevard, Nashville, TN 37208, USA; E-Mail: csharan@mmc.edu

**Keywords:** viral propagation arrest, λ phage, ectopic origins of DNA replication, DNA replication, DNA electron microscopy

## Abstract

The initiation step of DNA replication is the crucial determinant of proliferation in all organisms. This step depends on the specific interaction of DNA sequences present at origins of DNA replication and their cognate activators. We wished to explore the hypothesis that the presence of ectopic origin copies may interfere with proper genome duplication. Bacteriophage λ was used as a model system. To this end, the outcome of an infection of an *E. coli* strain harboring ectopic copies of the λ origin region was analyzed. By measuring the effect on the host growth, viral production, and electro-microscopic visualization of the resulting λ replicative intermediates, we concluded that the ectopic copies had prevented the normal initiation step of λ DNA replication. These results suggest that DNA decoys encoding viral origins could constitute effective tools to specifically arrest viral proliferation.

## 1. Introduction

The initiation of DNA replication constitutes the determinant step in the process of genome duplication. The replicon model, proposed more than fifty years ago [1] provided a practical framework for a molecular understanding of this step. According to this model, and in its most basic configuration, a replicon is a DNA segment whose duplication depends on the presence of a replicator (a DNA sequence, now named an origin of DNA replication, Ori), and an activator (a protein or proteins) whose function is to recognize the replicator, and to facilitate the recruitment of the replication machinery to this site, such that DNA synthesis could begin. In most simple organisms, such as bacteria and viruses, their genomes are constituted by a single replicon. Therefore, the binding of an activator protein to the unique Ori site is sufficient to initiate the duplication of their genomes. More importantly, it appears that even among closely related organisms both Ori and activators are unique. Thus, the exquisite interplay of these two critical elements determines the species-specificity of the initiation step, and of the duplication of the genome as a whole. It is, therefore, not surprising that in the simplest biological systems, such as viruses, the genomes, by providing just these two elements and by hijacking host proteins required for DNA, RNA, and protein synthesis, ensure their specific propagation.

Given these considerations one could predict that the presence of ectopic copies of a viral Ori region would interfere with proper viral propagation. In this report, we have decided to test this idea by using bacteriophage λ infection of *E. coli* cells as a model system.

Initiation of replication of λ DNA is dependent on the presence of a specific DNA sequence at the origin of replication, λOri, and of two phage proteins encoded by the genes *O* and *P*, since mutants defective in any of these elements fail to replicate in a detectable way [2,3]. Also supporting this view, a λ DNA segment carrying only the *O* and *P* genes, λOri, the promoter p_R_, and the *cro* gene, is capable of autonomous replication as a plasmid (λdv) [4,5]. The λOri region, which is curiously located within the O gene, contains four nearly perfect repeats of a 19 bp sequence which are separated by a few base pairs, and an adjacent 40 bp segment to the right of the repeating units which is rich in A-T pairs. Both of these two λOri elements are important for Ori function. The product of the phage genes O and P are involved in the initiation of λ DNA replication since addition of purified O and P proteins is sufficient to promote the replication of a λdv plasmid *in vitro.* [6]. However, only the O protein interacts with λOri. Moreover, the binding of O protein to λOri is very specific since other lamboid phages cannot complement λO^−^ mutants [7]. While this interaction represents the first step in the initiation of λ DNA replication [8], the P protein and other host proteins also participate in the initiation step. There is genetic and biochemical evidence that O and P proteins interact, and that P interacts with several host proteins [2]. The conjunction of all these activities leads to the recruitment of a helicase around the activated λ Ori region.

The O protein specifically interacts with the four 19 bp iterons in λOri through its amino-terminal half [6,8]; multiple copies of the O protein are involved [9]; and the binding causes a distortion at λ Ori [10,11,12,13]. As stated earlier, the λOri is located within the O gene; however, several mutants among them the r99 and ti12 variants used in the present study while impairing λOri function do not affect O protein activity. *In vitro* studies with purified O protein, and a supercoiled plasmid DNA containing λOri as a substrate, indicated that the binding of O to λOri causes a DNA distortion that makes the DNA region around the A-T rich element of λOri sensitive to S1 nuclease digestion suggesting that the O protein is involved in causing localized unwinding of the DNA at this region [10]. The same study showed that the comparative binding of O protein to the ti12 and r99 λOri variants followed the order r99 < ti12, which correlated with their sensitivities to S1 digestion [10].

To test our hypothesis that ectopic copies of λOri may interfere with a normal λ infection, we determined the bacterial growth profile, as well as viral proliferation after λ infection of *E. coli* containing a multicopy plasmid harboring the λOri region. Our results, coupled with analysis of the replicative intermediates resulting from λ infection indicated that indeed λ proliferation was arrested by the presence of ectopic λOri copies in the infected cell, possibly by interfering with the interaction of the λOri site and the initiator O protein, a requirement for the proper initiation of λ DNA replication.

Our findings suggest that the uniqueness of the replication initiation of viral DNAs, which ensures the specific propagation of their genomes, also represents a potential “Achilles heel” for these organisms, since ectopic copies of their Ori region could potentially be used as decoys to arrest their replication.

## 2. Materials and Methods

### 2.1. Bacterial, Plasmids, and Phage Strains

*E. coli* K12 strains used in this study, with the genotype shown in parenthesis were: CR34 (thr, leu, thy); and HBT, a thy^−^ derivative, of HB101(recA13, leu, pro). HBT(pOri1), HBT(pOrir99), and HBT(pOriti12) were constructed by transformation of HBT with the respective plasmid DNA using conventional methodology. Plasmid copy number has been estimated by others and us to be in the range of 20–40 copies/cell (Sigma-Aldrich Corp., St Louis, MO, USA; [14]). Bacteriophage λcII_68_cIII_67_, a virulent strain of phage λ, was used in all our experiments. ^3^H-labeled λcII68cIII67 DNA was harvested from agar plates seeded with infected bacteria and ^3^H methyl thymidine as previously described [15]. Radioactive phage was pelleted by centrifugation and suspended in 0.01 M M Tris pH 7.4, 0.01 M MgSO_4_ (TM) in ^2^H_2_O. Unlabeled λcII68cIII67phage was prepared in the same manner, but in the absence of radiolabeled thymidine.

### 2.2. Bacterial Growth and Phage Proliferation Studies

*E. coli* HBT and its derivatives were grown in a dry shaker at 37 °C in LB medium. The growth of the culture was monitored at selected time points by measuring the turbidity of the culture at 600 nm. The growth of infected cultures where bacteriophage λcII_68_cIII_67_ had been added at a moi = 5 or at a selected moi, was also monitored the same way. To assess phage proliferation, aliquots from infected cultures were taken at selected time points and the titer of the phage determined by standard procedures [16].

To determine the effect of addition of HBT(pOri1) to HBT infected cultures, HBT was infected at moi = 0.1. After placing the infected cultures at 37 °C in LB medium, HBT(pOri1) corresponding to one tenth of the initial HBT density was added and both the growth and phage yield of the mixed culture monitored at selected time points as described above.

### 2.3. Isolation of Intracellular λ DNA

The procedure described by Valenzuela and Inman [17] was followed. Briefly, 3 mL of HBT (or HBT(pOri1)) grown overnight in ^2^H_2_O-maltose medium was diluted 10-fold in the same medium and supplemented with ^15^N-containing algal hydrolysate plus thymidine. At OD_600_ = 0.6 the culture was pelleted by centrifugation, washed with 15 mL TM buffer in ^2^H_2_O, and resuspended in 1 mL of the same solution. Cells were left at 37 °C for 30 min. and then infected with radiolabeled λ at moi = 5. After adsorption at 37 °C for 15 min, the mixture was poured into a warmed 10 mL of ^2^H_2_O-Glucose medium supplemented with ^15^N-containing algal hydrolysate and thymidine. After further incubation at 37 °C for 12 min, the cultures were rapidly poured over 12 mL of a solution of cold 30% (*v/v*) pyridine in 0.03 M KCN that had been previously poured over 8 gr of crushed ice. After 2 min at 4 °C, the culture was centrifuged and the pellet resuspended in 1.5 mL of 0.03 KCN. Thirty microliters of freshly prepared 50 mg/mL lysozyme was then added. The suspension was the frozen and thawed 3× with cold water, followed by an incubation at 4 °C for 20 min. Thirty microliters of 30% sarkosyl was then added followed by an incubation at 4 °C for 20 min at 51 °C for 15 min. Finally, the lysate became clear by the addition of 100 μL of 20 mg/mL pronase in 5 mM EDTA pH 7 followed by an incubation with slow shaking at 37 °C for 2 h.

### 2.4. Detection of Intracellular λ DNA by CsCl Equilibrium Density Gradient Centrifugation

The clear lysate from above was made to have a density of 1.67 by mixing 4.6 gr of CsCl with 3.9 mL of lysate. The mixture was then used to fill a 6 mL polyallomer tube and run at 40,000 rpm for 17.5 h in a 60 VTi rotor. After the run the sample was fractionated from the top using an ISCO density gradient fractionator (Model 159, ISCO, Inc., Lincoln, NE, USA) into about 0.2 mL per fraction to yield a total of 30 fractions. Twenty-five microliter aliquots from each fraction were placed on filter discs. Filters were dried under a light lamp then about 50 μL of 10% TCA were added, dried, and washed twice with 0.25 N HCl, and once with 95% ethanol, and ether anhydrous. The filters were then placed in scintillation vials, about 6 mL of scintillation fluid added, and after vigorous mixing the vials counted in a scintillation counter for 2 min. The amount of radiolabeled DNA in each fraction was then plotted to determine the shift in density of the infecting LL DNA. Under our experimental condition LL DNA should be present around the middle of the gradient (fractions #15–16). Fractions containing DNA at densities between LL and HL were pooled and subjected to a second CsCl gradient centrifugation. Thirty microliters of selected fractions in the LL-HL region were then dialyzed against 1L NaCl/EDTA (20 mM/5 mM, pH 7) at 4 °C on Millipore filters for 4.5 h. An aliquot of the dialysate was then used for electron microscopy.

### 2.5. Electron Microscopy of DNA, Measuring, and Computation of Data

The methodology described by Schnӧs and Inman [18] was strictly followed, except that the micrographs were traced using a Numonics graphics calculator (Numonics Corp., North Wales, PA, USA).

### 2.6. RNase Treatment of λ Replicative Intermediates

Fractions containing D-loop structures were chosen for this treatment. The procedure described by Chattoraj and Stahl (20) was followed. Briefly, seven microliters of the selected fraction was treated with 1.5 units of RNaseH (Promega Corp., Madison, WI, USA) in 4 μL of a buffer containing 20 mM Hepes KOH, pH 8.0; 10 mM MgCl_2_; 50 mM KCl; and 1 mM DTT. The mixture was incubated for 20 min at 37 °C. The sample was dialyzed on Millipore filters as above, and an aliquot of the dialysate used for electron microscopy analysis.

## 3. Results

### 3.1. *E. coli* Harboring a Plasmid Containing the λ Origin of DNA Replication is Resistant to λ Infection

**Figure 1:**
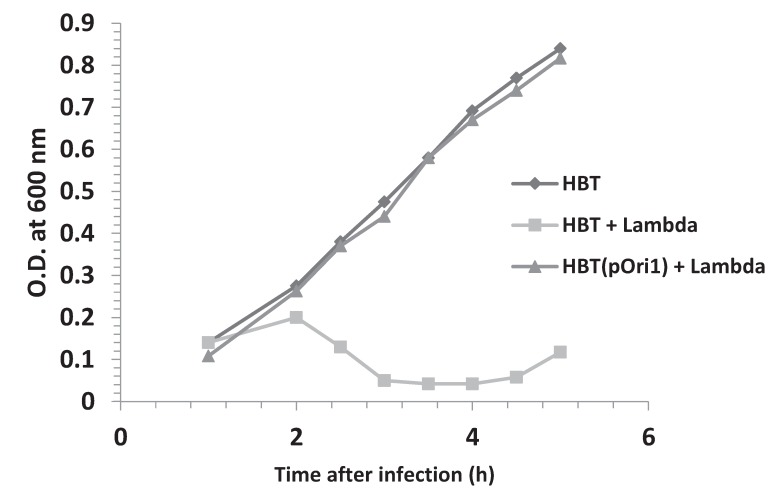
Effect of harboring ectopic copies of λOri on the growth profile of HBT after λ infection. The turbidity at OD_600nm_ of HBT, an *E. coli* K12 thy^−^ derivative of HB101(recA13, leu, pro), and that of its derivative HBT(pOri1) harboring the plasmid pOri1, were determined at indicated times following λ infection. The growth profile of an uninfected HBT culture was also determined and is shown as a control.

It has been well established that initiation of DNA replication in bacteriophage λ depends on the presence of a single viral origin of DNA replication (λOri), which serves as a landing pad for the formation of a DNA-protein complex that facilitates the recruitment of a helicase around the λOri DNA region [2,7]. λOri is located within the DNA sequence encoding the O protein, an important player in the initiation of λ DNA replication. The minimum λOri region is contained within a 164 bp sequence that consists of two parts: Four 19 bp repeating units, (iterons I–IV, separated from one another by short spacers), on the left; and a 49 bp region on the right (next to iteron I), which is A-T rich and highly strand asymmetric [19]. Both parts of the λOri region are required for replication initiation as demonstrated by the behavior of Ori^−^ mutations in both segments [20,21]. To test if an ectopic λOri, contained in a pBR322-derived recombinant plasmid named pOri1 [10], could interfere with this interaction during a normal λ infection, we first compared the growth profiles of isogenic bacterial *E. coli* strains containing or not pOri1, following λ infection. The results shown in Figure 1 indicate that upon λ infection, while the growth profile of the strain lacking pOri1 was typical of a lytic infection, the strain containing pOri1 grew as if had not been infected at all by the virus. In fact, its growth profile was similar to that of an uninfected culture. These results suggested that the presence of pOri1 somehow protected the bacteria from a lytic infection. To ascertain that this resistance was dependent solely on the presence of the λOri region, bacteria containing only the parental plasmid were infected under the same conditions as above. As expected, the resulting growth profile was indistinguishable from a normal lytic infection (Figure S1). We also investigated the growth profile of an isogenic bacterial strain harboring two ectopic copies of the λOri region (pOri2). We did not notice any significant difference between the strains harboring one or two copies of the λOri region (Figure S2). This result suggested that one ectopic copy of the λOri region per plasmid is sufficient to provide resistance to λ infection.

### 3.2. Phage Proliferation Is Impaired in Bacteria Harboring Ectopic Copies of λOri

**Figure 2:**
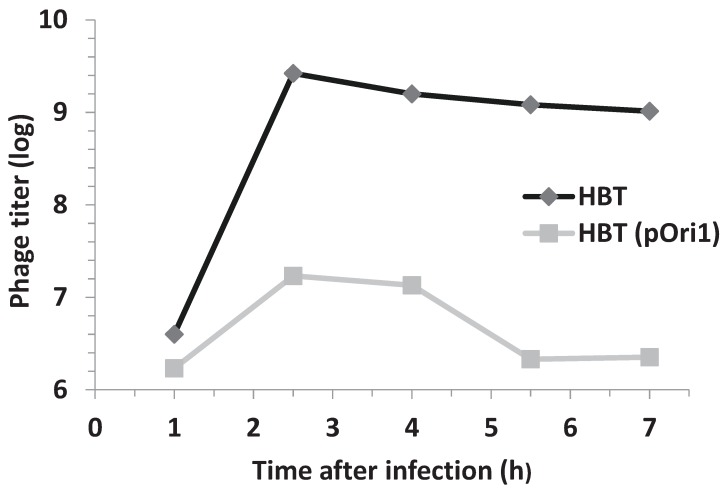
Phage titers following λ infection of HBT and HBT(pOri1). The phage titer from λ-infected cultures of HBT (pOri1) or HBT were determined at indicated times after infection, by plating dilutions of the culture unto plates containing sensitive bacteria HBT cells. Titers are expressed in a log scale.

**Figure 3:**
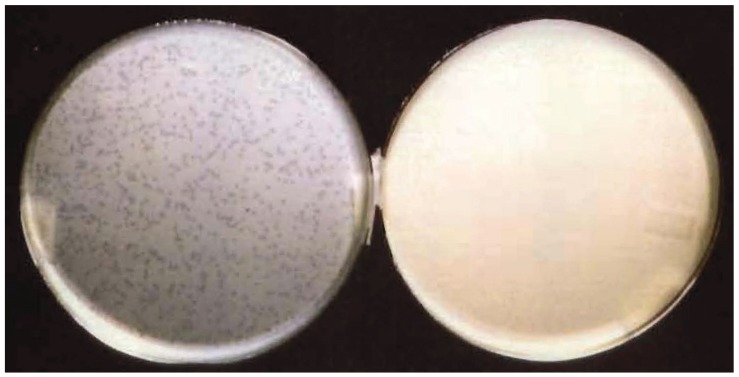
λ infection of HBT and HBT(pOri1) on agar plates. About 1000 λ particles were tittered on agar plates containing either HBT (left plate) or HBT(pOri1) (right plate).

The *E. coli* strain used in our experiments is a suitable host for λ infection. To investigate if its resistance to λ infection was due to an impairment in phage proliferation we measured the phage yield in the strain lacking or harboring the recombinant plasmid pOri1. To this end cultures of *E. coli* HBT or HBT(pOri1) were infected with phage λ at moi = 5 and the yield of phage particles determined at different time points after infection. The results shown in Figure 2 indicated that phage production was almost completely arrested in the HBT(pOri1) strain whereas the yield in the HBT strain was as expected of a lytic infection. The yield difference found between the two strains was about three orders of magnitude. This proportion was confirmed when about 1000 particles of phage λ were used to infect either HBT or HBT(pOri1) and plated. As shown in Figure 3, it is evident that while the titer on HBT was as expected, no viral plaques were observed on the plate containing HBT(pOri1). These results indicate that it is the inability of the infecting phage particles to proliferate in bacteria harboring ectopic copies of the viral origin of replication that leads to the bacterial resistance to lytic infection.

### 3.3. Effect of Multiplicity of Infection and λOri Variants on Bacterial Resistance

**Figure 4:**
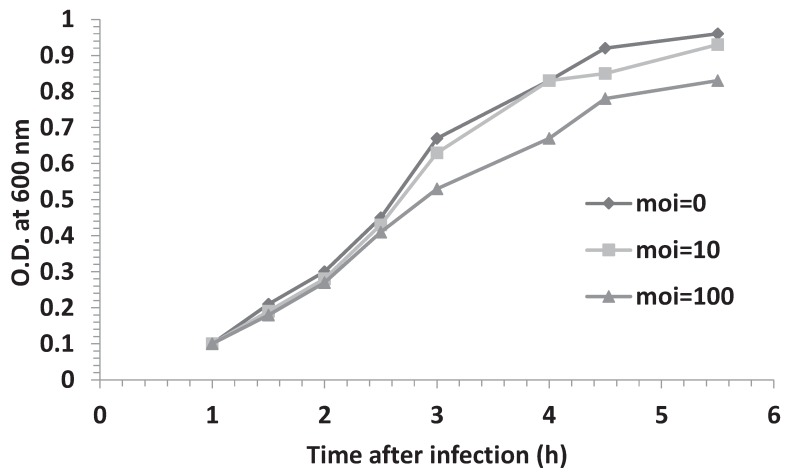
Effect of moi on the growth profile of infected HBT(pOri1). HBT(pOri1) cultures were infected at moi = 10 or 100 and the bacterial growth monitored at the indicated. The growth of an uninfected culture (moi = 0) was monitored as control.

The experimental set up of all our previous studies involved infecting bacterial cells at a multiplicity of infection (moi) equal to five. Given the estimation that each HBT(pOri1) cell contain about 20 to 40 copies of the plasmid pOri1 (see Materials and Methods section), our results suggested that at this moi, phage DNA molecules may be in numerical disadvantage. We, therefore, decided to test if bacterial resistant depended on the moi by determining the growth profile of HBT(pOri1) cultures that had been infected at mois of 10 and 100, respectively. Our data shown in Figure 4 indicated that at moi = 10 the cultures yielded a growth profile that was similar as that previously observed at moi = 5. Only at moi = 100 do we noticed a minor change in the growth profile of the infected culture. We have also tried using mois lower that 5, with results that were undistinguishable from uninfected cultures. These results indicated that the overall number of ectopic copies of the λ origin in HBT(pori1) were sufficient to arrest phage proliferation.

**Figure 5:**
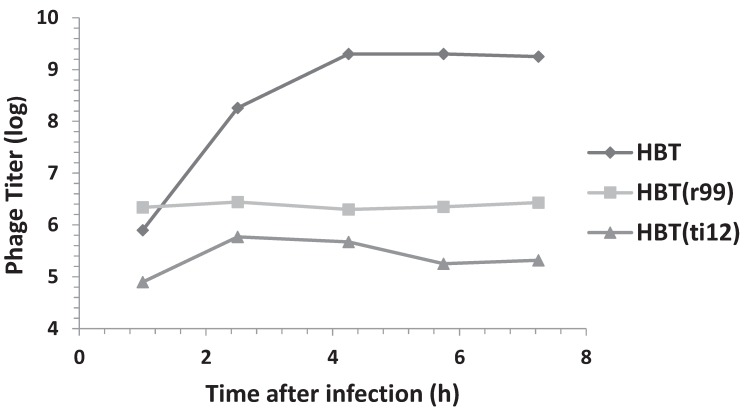
Phage titers following λ infection of HBT, and the HBT(pOri1) variants r99 and ti12. The phage titer from λ-infected cultures of HBT and HBT (pOri1) variants r99 and t1t12 were determined at indicated times after infection, as previously described. Titers expressed in a log scale.

Several mutants within the λOri region have been described [19,20]. These mutants have the common effect of impairing the efficiency of λ proliferation. Two of these mutants, r99 and ti12 have been well characterized and both reside in the A-T rich arm of the λOri region. The r99mutant consists of a 12 bp deletion located immediately to the right of the iteron I. The ti12 mutation is a CG > AT transversion that is contained in the 12 bp segment removed by the r99 deletion [19]. In both of these mutants, λ proliferation was found to be affected at least 10 fold, the effect being stronger in the r99 mutant [20]. Interestingly, *in vitro* studies showed that the binding of these λOri mutants to the λ O protein, compared to the wild type λOri, was still significant (35% for r99 and 49% for ti12, respectively [10]). Given that the variants of the recombinant plasmid pOri1 containing each of the mutants (pOrir99 and pOriti12, respectively) were available [10], we decided to compare the effect of ectopic copies of these variants on bacterial resistance, and phage production following λ infection. As with HBT(pOri1), the variants exhibited resistance to λ infection. Additionally, as shown in Figure 5, the phage titer profile of the infected variants was significantly lower than that found in the control HBT strain. We noticed however that the pOriti12 variant appeared to be more resistant that its pOrir99 counterpart. Altogether, these findings support the view that the binding capacity to the O protein alone (which is not arrested in these variants), rather than the presence of a native λOri region, may be sufficient to cause both the λ resistance, and the diminished phage proliferation phenotypes.

### 3.4. Addition of HBT(pOri1) to λ-Infected HBT Relieves the Sensitivity of HBT to λ Infection

**Figure 6:**
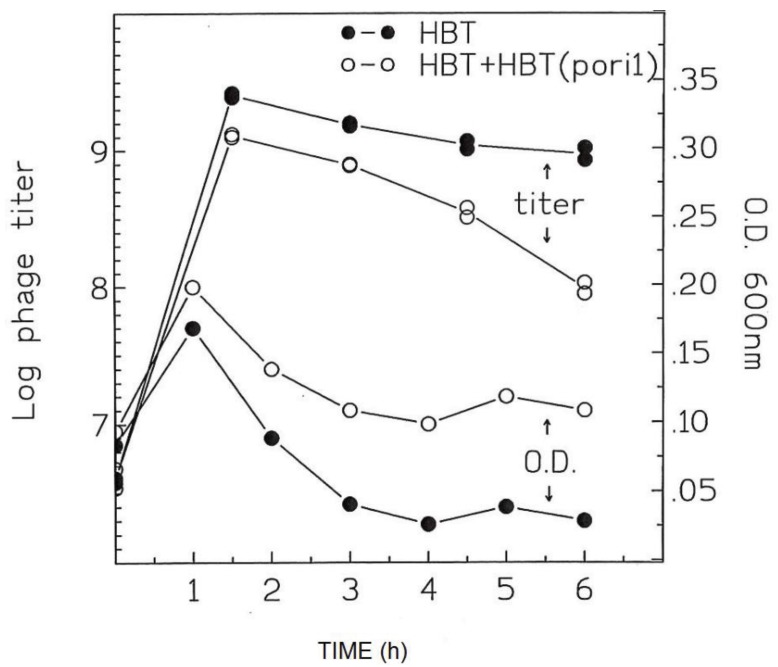
Effect of addition of uninfected HBT(pOri1) culture to a λ-infected HBT, on bacterial growth and *phage titer.* A culture of λ*-infected HBT* was either left alone (HBT) or supplemented with a 1/100 volume of uninfected HBT(pOri). Both the turbidity of the cultures, and the phage titers, were determined at the indicated times after infection (phage titers are expressed in a log scale).

Since HBT(pOri1) is resistant to λ infection, we reasoned that the addition of HBT(pOri1) cells to a culture of infected HBT, may aid in prolonging the survival of HBT cells. To this end, we infected a culture of HBT with λ at a moi = 0.1 and HBT(pOri1) was added at a ratio of 1/100 of the HBT culture. A various times, both the bacterial growth and the phage titer were monitored in the mixed culture batch. As a control, the profile of HBT infected with lambda was also determined. As shown in Figure 6, the growth of the mixed culture improved compared to the control culture. More interesting, a significant drop in the phage titer was also observed as the growth of the mixed cultures progressed. These results suggested that HBT(pOri1) was capable of rescuing HBT from further λ infection by sequestering λ particles into a non-productive viral outcome, thus allowing non-infected HBT to proliferate.

### 3.5. Replicative Intermediates Found in λ-Infected, HBT(pOri1) or Variant Strains, Are Reminiscent of Intermediates Isolated from λO^−^ Mutants

The observations presented above suggested that the resistance of HBT harboring pOri1 or its variants resulted from an impairment of phage proliferation. Moreover, the fact that the λOri variants (which when located in the λ genome affect its replication), had retained their capacity to bind to the O protein [10], provided us with the hypothesis that the tittering or competitive binding of the ectopic copies of the λ origin region to the O protein, produced by the infecting viral DNA, inhibited the initiation step of DNA replication, similar to that observed in λ initiation mutants [22]. The most straightforward way of determining the manner by which DNA replication occurs in small genomes is by the visualization of its replicative intermediates in the electron microscope. For bacteriophage λ, this technique, coupled with partial denaturation, has been instrumental in determining several crucial features about λ DNA replication [17,18,22,23,24,25,26,27]. We therefore decided to use these combined techniques to analyze the replicative intermediates arising after λ infection of HBT and HBT(pOri1), respectively, during the early stage of λ DNA replication.

In order to isolate λ replicative intermediates, bacteria is grown for several generations in a “heavy” medium (where H_2_O has been replaced by D_2_O, and ^14^N-containing nitrogen source replaced by its ^15^N isotope), such that its doubling time is delayed about six-fold, and its DNA has a heavier density (HH) than normal due to the incorporations of both ^2^H and ^15^N atoms. When radioactively labeled phage grown under normal condition (LL) infects the bacteria in “heavy” medium its DNA acquires the heavy isotopes during its replication leading to an increase in the intrinsic density of its DNA. At early stages of replication the resulting viral DNA can be analyzed through two successive cycles of CsCl equilibrium gradient centrifugation and found to be localized between the densities corresponding to LL and HH DNA [26]. The extent of migration of the viral DNA toward the heavier density is an indication of the extent of DNA replication in the “heavy” medium. Therefore, the profile of the second CsCl gradient usually serves an earlier predictor of the extent of viral DNA replication.

**Figure 7:**
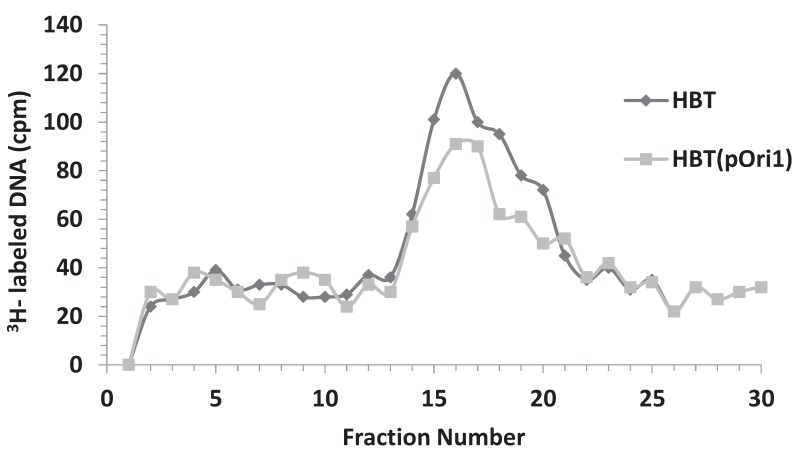
DNA fractionation after a second CsCl equilibrium density centrifugation of intracellular ^3^H-labeled λ DNA isolated from infected HBT or HBT(pOri1). CsCl fractions containing viral DNA, and separated from bacterial DNA through a first CsCl gradient, were pooled and run through a second CsCl in order to isolate newly synthesized DNA. At the end of the run, DNA was fractionated and the radioactivity in each fraction, from the lowest density fraction (fraction #1) to the highest density (fraction #30) was determined by scintillation counting. Under the conditions of centrifugation the density of LL DNA is around fractions 15–16. Note that the DNA shoulder past fraction 16 is more prominent in HBT compared to HBT(pOri1).

**Figure 8:**
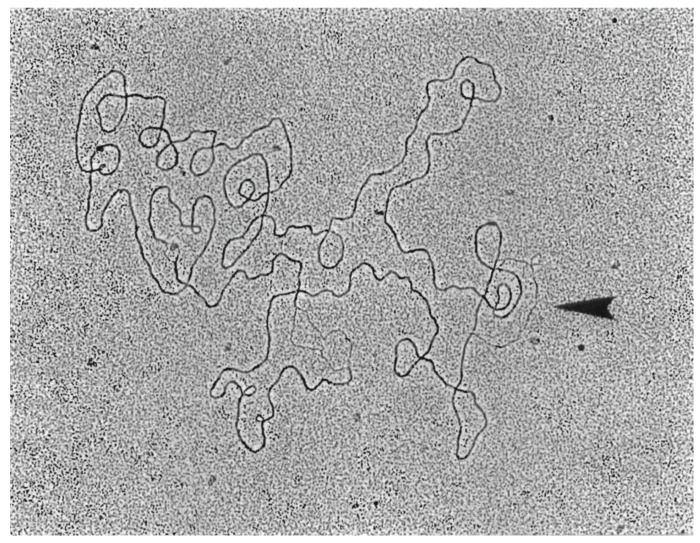
Electron micrograph of a representative λ circular DNA containing a D-loop structure. Electron micrograph of a circular λ DNA molecule showing a region containing a D-loop (indicated by the arrow) obtained from a λ-infected HBT(pOri1) culture. Thick line represents double stranded DNA, whereas thin line, single stranded. Note that the circular DNA shown also has two regions undergoing denaturation (bubble like structures containing two opposite thin lines), a normal occurrence during DNA spreading. These regions are different from the D-loop since this latter structure shows a thick line (double stranded DNA segment) opposite to a thin one.

When λ DNA was isolated from infected HBT or HBT(pOri1) and purified through a second CsCl gradient, it was apparent that the intracellular λ DNA obtained after HBT infection was slightly more skewed toward heavier CsCl densities compared to the one obtained from the infected HBT(pOri1) strain (Figure 7). This suggested that the extent of λ DNA replication in HBT had been comparatively greater than in HBT(pOri1). When selected fractions (between DNA densities corresponding to LL and HL) from these two preparations were analyzed by electron microscopy, about 60% of all λ DNA structures observed (*n* = 138) in infected HBT represented normal theta replicative intermediates [9,10], whereas in HBT(pOri1) only 8% of all structures (*n* = 107) belonged to this category. These results confirmed our prediction that in HBT(pOri1) λ DNA replication had been impaired. More importantly, in HBT(pOri1) about 66% of all the λ DNA molecules observed contained a “D-loop” like structure (a representative molecule is shown in Figure 8). In contrast, no such structures were observed in preparations obtained from infected HBT. D-loops had been typically observed in λ mutants (such as O and P gene mutants) that were defective in the initiation step of DNA replication [22]. Treatment of these structures with RNaseH caused the disappearance of these D-loops indicating that one of the strands in the loop was made up of RNA [8]. These structures, also found under replicative stress conditions [27] have been interpreted as DNA molecules attempting to use transcription to initiate DNA replication, Supporting this view is the finding that the location of the D-loops in λO^−^ mutants occurs around highly transcribed regions [22]. To ascertain that the “D-loop” structures we had observed after λ infection of HBT(pOri1) had a similar composition we subjected our samples to a similar RNAseH treatment. Electron microscopic observation of the resulting DNA samples yielded no loop structures (*n* = 137) demonstrating that DNA structures we found also contained RNA at the “D-loops” thus furthering the similarity between the outcome of λ infection of HBT(pOri1) and λO^−^ infection of *E. coli*.

**Figure 9:**
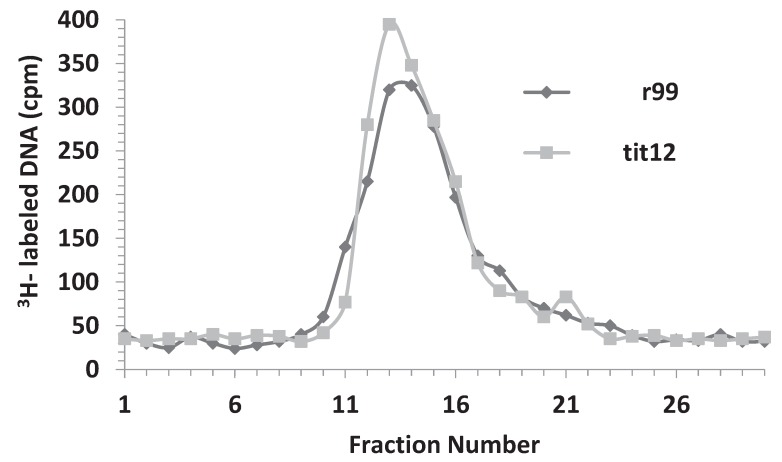
DNA fractionation after a second CsCl equilibrium density centrifugation of intracellular ^3^H-labeled λ DNA isolated from infected HBT(pOrir99) or HBT(pOriti12). The density profile of intracellular λ DNA was obtained as described in Figure 7. Note that the profiles obtained from both of the infected strains are indistinguishable from each other.

The intracellular DNA isolated from infected HBT strains harboring the λpOri1 variants, pOrir99, and pOriti12 respectively, was also analyzed in a similar manner. We found that the second CsCl profile of the λ infected strains containing these variants, was similar to that obtained with infected HBT(pOri1). Figure 9 shows that the profile of the second CsCl gradient for the variants was indistinguishable from each other. Moreover, electron microscopic inspection of the intracellular λ DNA showed that also in these strains a significant number of molecules were observed to contain D-loops (55 out of 112 for the ti12 variant, and 40 out of 116 for the r99 variant, respectively). Finally, treatment of these fractions with RNaseH also abolished the D-loop structures.

The results of our electron microscopy studies support the conclusion that the presence of ectopic λOri copies in the λ-infected cell reduced the level of the O protein, such that the λ infection in these cells had the same outcome as that obtained with normal cells infected with a λO^−^ mutant. In this context our work with the λOri variants, r99 and ti12, was consistent with *in vitro* binding studies reported elsewhere indicating that these mutants had retained O-binding activity [10]. Our work showed that even with this reduced binding activity (35% for r99, and 49% for ti12, respectively [10]), they were capable of arresting the replication of the incoming phage. It is also interesting to note that the proportion of D-loops found with these variants also correlated with their degree of O-binding [7].

## 4. Discussion

According to the replicon model proposed more than 50 years ago [1] the interplay of origins and initiator(s) determines the opening of DNA strands for a replicon to become a substrate for initiation of DNA synthesis. Origins are DNA sequences that can be recognized by species-specific proteins, and in most viral and bacterial systems origins are defined by unique DNA sequences, which are targeted by their cognate initiator protein.

In this paper we have investigated the effect on cell survival to viral infection when a bacteria harbors ectopic copies of a viral origin of DNA replication. As a proof of principle we have used the well-documented infection cycle of *E. coli* by bacteriophage λ.

Our results viewed in the context of the interaction of the O protein with λOri, validated our hypothesis that the presence of extra λOri copies in the host bacteria competed with the viral sequence for the recruitment of O protein leading to an arrest of proper viral proliferation. This implies that the O protein is able to function *in trans* and that the large copy number of plasmids in each bacterium denied the incoming λ phage from establishing an efficient initiation complex required for DNA replication. This conclusion is supported first, by the initial observation that HBT(pOri1) was resistant to λ infection, and that the phage yield in this strain was considerably lower than in HBT; second, the growth resistance and low phage yield phenotypes could also be observed if the infected sensitive strain (HBT) is mixed with the resistant one; third, analysis of the intracellular λ DNA from obtained HBT(pOri1) infected cells, by electron microscopy, showed a dramatic reduction in the number of normal replicative intermediates, and the occurrence of D-loop containing DNA structures, identical in structure and composition to those obtained from *E. coli* infected with λO^−^ mutants; finally, our studies with the λOrir99 and λOriti12 variants, which yielded similar results as with λOri1, are in agreement with our hypothesis since in these mutants O binding, albeit reduced, is not abolished [10].

In summary, our studies have exploited the exquisite interaction between an origin of DNA replication and its cognate activator to demonstrate that this interaction can constitute a potential viral “Achilles heel” which can be targeted in viral eradication programs in diseases of human importance. It has also not escaped our attention, that the strategy we have used in this study could also be applied to prevent the proliferation of viral DNA that has been integrated into the host genome. To our knowledge, this study explored a novel approach of specifically targeting the duplication of a genome by using ectopic copies of its origin of DNA replication as decoys.

## 5. Conclusions

In the present study we have explored the possibility of using ectopic copies of origins of DNA replication to interfere with the proper duplication of a viral genome. This approach, as applied to the bacteriophage λ system, resulted in the arrest of the early stage of λ DNA replication, suggesting that ectopic origins of DNA replication could be used as decoys to prevent the formation of the protein-DNA complex required to initiate DNA replication.

## Supplementary Files

Supplementary File 1
