# Supplementary material for: Arrest of Viral Proliferation by Ectopic Copies of Its Cognate Replication Origin
Source: Genes (Basel). 2015 Jun 23;6(2):436–50. doi: 10.3390/genes6020436 (PMC4488673; doi:10.3390/genes6020436)
Supplement: Supplementary File 1 [file genes-06-00436-s001.pdf]

Supplementary Materials

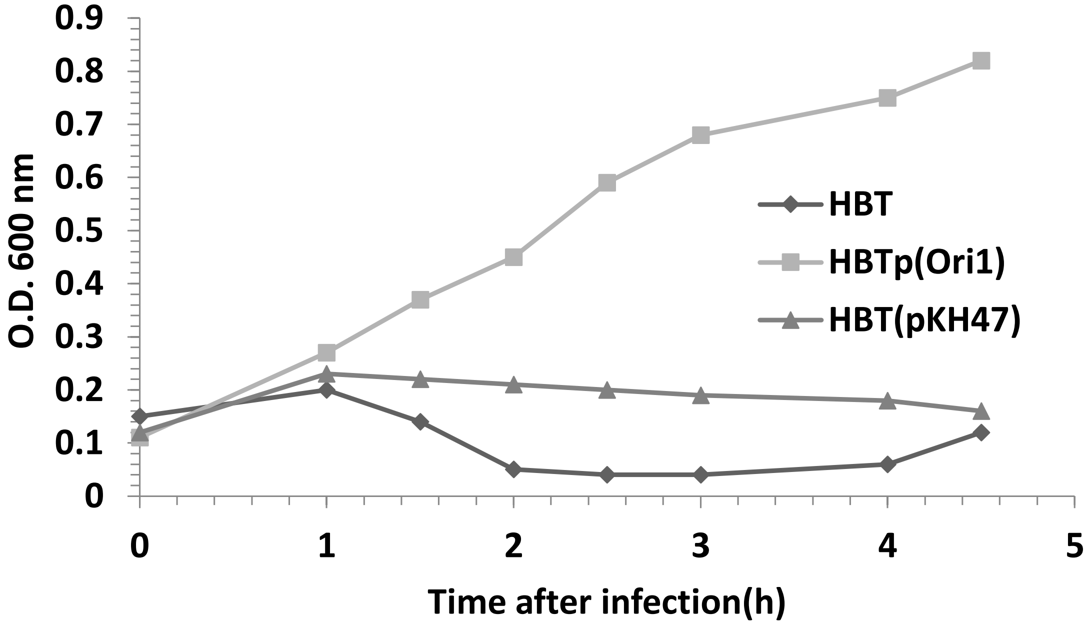

Figure S1. G*rowth of λ-infected HBT containing a plasmid lacking the λOri region*. HBT containing pKH47, a closely related plasmid to pOri1, but lacking the λOri region, was infected with λ phage, and its growth profile monitored and compared to a λ-infected HBT(pOri1), and to an uninfected HBT.

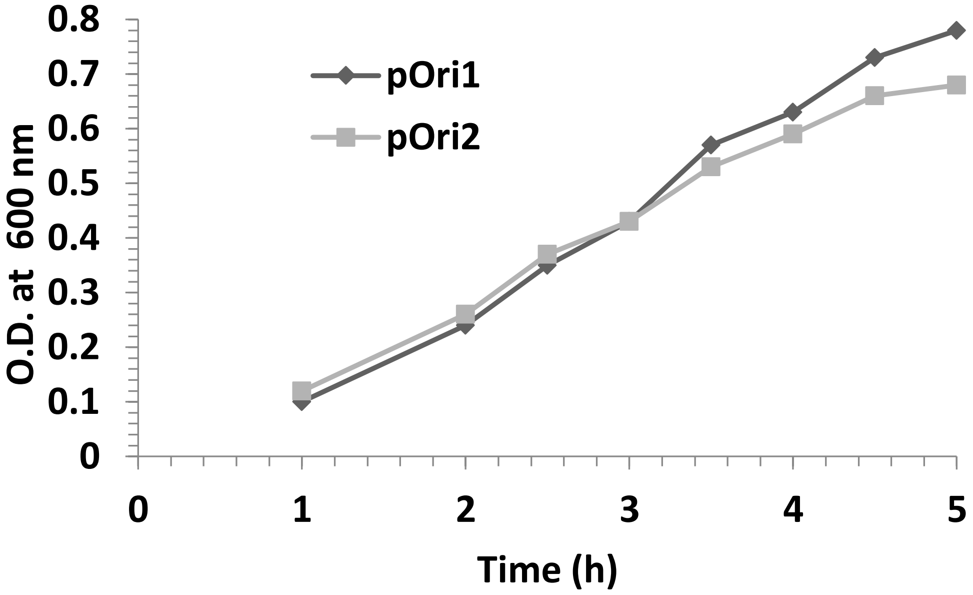

Figure S2. *Effect of harboring two copies of the λ Ori region per plasmid on the growth profile of HBT after λ infection.* HBT(pOri2) a strain containing, a variant of plasmid pOri1, harboring two tandem copies of the λOri region was infected with λ, and its growth profile monitored and compared to a λ-infected HBT(pOri1), and to an uninfected HBT.
